# Associations Between Social Engagement and Physical Activity Among Community-Dwelling Older Adults With Dementia

**DOI:** 10.1093/geroni/igaa057.905

**Published:** 2020-12-16

**Authors:** Aya Yoshikawa, Matthew Smith, Marcia Ory

**Affiliations:** Texas A&M University, College Station, Texas, United States

## Abstract

People with dementia (PWD) tend to experience loneliness and physical inactivity, which increases their risk of negative health outcomes such as depression and declined physical functioning. Research indicates the link between supportive social environments and physical activity (PA), yet this association is not fully studied among PWD. This cross-sectional study investigated associations between social engagement and moderate (i.e. walking for exercise) and vigorous PA among PWD. A subset sample of community-dwelling Medicare beneficiaries in the 2015 National Health and Aging Trends Study, representing PWD including possible (n = 779) and probable (n = 902) dementia, were analyzed using survey-weighted logistic regression analysis. All analyses were controlled for sociodemographic characteristics, living arrangement, physical functioning, and the number of chronic conditions. The majority of participants was 80 years or older (52.5%), female (53.1%), White (67.4%), had a high school or higher education (64.0%), and two or more chronic conditions (74.1%). Living alone (OR = 1.60, 95%CI 1.22, 2.10), knowing their community well (OR = 1.56, 95%CI 1.12, 2.16), and going out for enjoyment (OR = 1.93, 95%CI 1.37, 2.73) were associated with moderate PA. In addition to going out for enjoyment (OR = 2.40, 95%CI 1.65, 3.53), attending organized activities (OR = 1.97, 95%CI 1.15, 3.39) and working as a volunteer (OR = 1.63, 95%CI 1.04, 2.57) were associated with vigorous PA. Findings suggest that enjoyable activities and community familiarity may facilitate walking behavior among PWD. Integrating events and volunteer activities within communities is encouraged for supporting active living among PWD.

